# The influence of acute kidney injury on the outcome of Stevens–Johnson syndrome and toxic epidermal necrolysis: The prognostic value of KDIGO staging

**DOI:** 10.1371/journal.pone.0203642

**Published:** 2018-09-07

**Authors:** Tao Han Lee, Cheng-Chia Lee, Chau-Yee Ng, Ming-Yang Chang, Su-Wei Chang, Pei-Chun Fan, Wen-Hung Chung, Ya-Chung Tian, Yung-Chang Chen, Chih-Hsiang Chang

**Affiliations:** 1 Kidney Research Center, Department of Nephrology, Change Gung Memorial Hospital, Linkou branch, Taoyuan, Taiwan; 2 Graduate Institute of Clinical Medical Science, College of Medicine, Chang Gung University, Taoyuan, Taiwan; 3 Department of Dermatology, Drug Hypersensitivity Clinical and Research Center, Chang Gung Memorial Hospital, Taipei, Linkou and Keelung, Taiwan; 4 Clinical Informatics and Medical Statistics Research Center, College of Medicine, Chang Gung University, Taoyuan, Taiwan; 5 Division of Allergy, Asthma, and Rheumatology, Department of Pediatrics, Chang Gung Memorial Hospital, Taoyuan, Taiwan; 6 Division of Nephrology, Department of Medicine, Keelung Chang Gung Memorial Hospital, Keelung, Taiwan; National Yang-Ming University, TAIWAN

## Abstract

**Background:**

Stevens–Johnson syndrome (SJS), toxic epidermal necrolysis (TEN), and SJS/TEN overlap syndrome are severe drug-induced cutaneous adverse reactions with high mortality. Acute kidney injury (AKI) was a common complication in an SJS/TEN group and noted as an independent risk factor for mortality in patients with SJS/TEN. To determine whether AKI staging can predict the outcome of patients with SJS/TEN, we compared the discriminative power of an AKI KDIGO staging system with that of SCROTEN, APACHE II, APACHE III, and SOFA.

**Materials and methods:**

We retrospectively analyzed the data of 75 patients who were diagnosed with SJS, TEN, or SJS/TEN overlap syndrome at a tertiary care university hospital between January 1, 2011 and December 31, 2014. The baseline characteristics, biochemical analysis data, medication use, and outcomes of the patients were assessed, and the discriminative ability for predicting mortality was determined for each prognostic model.

**Results:**

Of the 75 patients, 23 (30.7%) had AKI, of whom 13 (56.5%) died during the index admission. Of the prognostic risk models analyzed, the KDIGO staging system showed similar discriminative ability in predicting in-hospital mortality as did the other models. In addition, combining KDIGO with other scoring systems yielded significantly more accurate risk prediction for in-hospital mortality compared with the other individual scores alone, as measured by net reclassification index. The patients with KDIGO stages 2 and 3 exhibited a significantly lower 1-year survival rate than did those with KDIGO stages 0 and 1.

**Conclusion:**

AKI KDIGO staging has good discriminative ability and is easy to utilize for predicting mortality.

## Introduction

Acute kidney injury (AKI) is a common, harmful complication with an incidence rate ranging from 28% to 75% in a hospital setting. [1–4] AKI-related adverse effects can potentially be controlled by early intensive intervention. Stevens–Johnson syndrome (SJS), toxic epidermal necrolysis (TEN), and SJS/TEN overlap syndrome are potentially fatal disorders characterized by high fever, widespread blistering exanthema of macules, and atypical target-like lesions, accompanied by mucosal involvement with a mortality rate of 10% for SJS and more than 30% for TEN. [5, 6] In a previous study, AKI was diagnosed in 20.8% of patients with SJS and TEN, with a 3.1% dialysis rate and a 5-times higher mortality rate.[7] Another study reported a higher prevalence of AKI among patients with SJS/TEN than among those without STS/TEN (odds ratio [OR] [95% CI] = 1.78, [1.36–2.33]). [8] Risk factors for SJS and TEN include human immunodeficiency virus, genetic factors, and malignancies. [7, 9–11] Hung et al. [7] reported sepsis, certain drugs (e.g., allopurinol, nonsteroidal anti-inflammatory drugs, and antibiotics), chronic kidney disease, and hypoalbuminemia as risk factors for AKI. Another study identified pre-renal azotemia and tachycardia as independent risk factors for death in patients with TEN. [12] However, outcome prediction based on the presence or the severity of AKI is not widely discussed in the literature. In 2012, the Kidney Disease Improving Global Outcomes (KDIGO) group modified the definition of AKI as follows: a 0.3-mg/dL increase in serum creatinine (SCr) within 48 hours, a 1.5-times increase in SCr from baseline within 7 days, or urine volume less than 0.5 mL/kg/h for 6 hours. The KDIGO group also indicated AKI severity based on changes in SCr and/or urine volume relative to the baseline condition. In this investigation, we analyzed different prognostic models and KDIGO score at the time of diagnosis and compared their ability in predicting the outcome of SJS/TEN patients.

## Materials and methods

### Study patients and design

This was a retrospective study performed using the data of patients who were diagnosed with SJS/TEN or DRESS syndrome in a tertiary care referral center in Taiwan between January 1, 2011 and December 31, 2014. Patients who were receiving dialysis, aged less than 18 years, or had received a prior organ transplant were excluded. Only one patient was excluded, specifically for being under 18 years old. The Institutional Review Board (IRB) of the study hospital approved the study and waived the need for informed consent due to the retrospective nature of the study, which did not compromise the privacy of any patients. The study protocol was approved by the IRB of Chang Gung Memorial Hospital in Taipei, Taiwan.

### Data collection and definitions

Diagnosis of the target diseases was confirmed by 2 dermatologists and the offending medication was further confirmed by a pharmacologist. We recorded the demographic characteristics, underlying diseases, biochemical analysis data, medications, and complications of the patients on the day of disease diagnosis. The Acute Physiology and Chronic Health Evaluation (APACHE) II, APACHE III, and Sequential Organ Failure Assessment (SOFA) scores as well as severity-of-illness score for TEN (SCORTEN)[12] were obtained based on data collected upon the index admission. Complications were recorded after the index day. Outcomes were investigated separately and included hospital stay, in-hospital mortality, and 1-year mortality. Post-hospital discharge data were retrieved by reviewing follow-up records. No cases were lost to follow-up during the study period. The diagnosis and severity of AKI were confirmed using the KDIGO Clinical Practice Guidelines for Acute Kidney Injury. [13] A simple model for classifying AKI severity was developed as follows: non-AKI (0 points), stage 1 (1 point), stage 2 (2 points), and stage 3 (3 points).

### Statistical analysis

The small sample size provided insufficient power to test the normality of continuous data. Therefore, continuous data were expressed as a median and interquartile range. The distribution of continuous and categorical data between the AKI and non-AKI groups was compared using the Mann–Whitney U test and Fisher’s exact test, respectively. The association of AKI with the risk of in-hospital mortality was evaluated using logistic regression analysis, in which several known risk factors were sequentially adjusted for. Due to the small sample size of this study, empirical estimates (e.g., those obtained using the Wald test) would be biased and the model would be overfitted. Therefore, we additionally performed logistic regression analyses by using the bootstrap percentile method with 5,000 samples to mitigate the small data bias. [14, 15] The discriminative ability of individual scores in predicting in-hospital mortality was determined using the area under the receiver operating characteristic curve (AUC). The AUCs of different scores were compared using a nonparametric approach. Optimal cutoff points were determined according to the Youden Index, and the corresponding sensitivity, specificity, positive likelihood ratio, and negative likelihood ratio were calculated. The calibration performance for each score was assessed using the Hosmer–Lemeshow goodness-of-fit test. The discriminative and predictive benefit of combining KDIGO with other scoring systems in predicting in-hospital mortality was assessed using the integrated discrimination index (IDI) and continuous (category-free) net reclassification index (NRI), respectively. [16, 17] Finally, we used the log-rank test to compare the 1-year Kaplan–Meier survival rates of the patients with KDIGO 0 and 1 with those of the patients with KDIGO 2 and 3. All statistical tests were 2-tailed and a *P* value of less than 0.05 was considered statistically significant. Data were analyzed using SPSS 22 for Windows (IBM Corp., Armonk, NY).

## Results

Seventy-five consecutive patients were assessed, of whom 23 (30.7%) had AKI and 15 (20%) died during admission. The baseline characteristics are shown in Table 1. Compared with the patients in the non-AKI group, those in the AKI group were older and had higher rates of chronic kidney disease and gout. The groups did not differ significantly in mean arterial pressure, leukocyte or platelet count, or bilirubin or sodium level. However, the AKI group exhibited lower levels of hemoglobin and albumin as well as poorer renal function and higher serum potassium levels. Moreover, the AKI group showed significantly higher SCROTEN, APACHE II, APACHE II, and SOFA scores.

**Table 1:** Baseline characteristics at SJS diagnosis, stratified by AKI Status.

Variable	All patients	AKI	Non-AKI	*P* value
Patient number	75	23	52	-
Age, y	64 (31)	75 (14)	58 (33)	< 0.001
Male sex, n (%)	34 (45.3)	10 (43.5)	24 (46.2)	1.000
Underlying disease, n (%)				
Diabetes mellitus	27 (36.0)	12 (52.2)	15 (28.8)	0.069
Chronic kidney disease	17 (22.7)	15 (65.2)	2 (3.8)	< 0.001
Chronic liver disease	6 (8.0)	2 (8.7)	4 (7.7)	1.000
Cancer/hematologic malignancy	8 (10.7)	5 (21.7)	3 (5.8)	0.053
Gout	9 (12.0)	7 (30.4)	2 (3.8)	0.003
Mean arterial pressure, mmHg	95 (26)	88 (31)	97 (24)	0.095
APACHE II	8 (7)	14 (7)	7 (3)	< 0.001
APACHE III	28 (30)	53 (25)	22 (15)	< 0.001
SOFA	1 (3)	4 (4)	1 (2)	< 0.001
SCORTEN	2 (1)	3 (1)	2 (1)	< 0.001
Lab data				
Leukocyte count, 1000/mL	8.0 (5.3)	8.9 (5.9)	7.5 (4.9)	0.260
Hemoglobin, g/dL	12.6 (3.3)	9.4 (3.7)	13.0 (2.2)	<0.001
Platelet count, 1000/mL	185 (100)	197 (100)	185 (86)	0.662
Bilirubin, mg/dL	0.5 (0.4)	0.5 (0.4)	0.5 (0.5)	0.917
Creatinine, mg/dL	0.92 (1.13)	2.25 (2.38)	0.73 (0.48)	<0.001
BUN, mg/dL	17.0 (25.7)	53.2 (38.3)	11.2 (8.6)	<0.001
Albumin, mg/dL	3.3 (1.0)	2.7 (1.0)	3.5 (0.7)	<0.001
Sodium, mg/dL	137 (5)	137 (6)	137 (5)	0.416
Potassium, mg/dL	4.2 (0.7)	4.5 (1.0)	4.0 (0.7)	0.020

Continuous data are presented as median (interquartile range).

AKI, acute kidney injury; APACHE, Acute Physiology and Chronic Health Evaluation; BUN, blood urea nitrogen; SCORTEN, severity-of-illness for toxic epidermal necrolysis; SJS, Stevens–Johnson syndrome; SOFA, sequential organ failure assessment score.

Disease etiologies, details, and complications during hospitalization are presented in Table 2 along with patient outcomes. The patients with TEN showed a higher prevalence of AKI than did the patients with the other 2 diseases. The most frequently induced skin hypersensitivity reaction drug was allopurinol (32%), followed by phenytoin (13.3%) and carbamazepine (8%). The patients with AKI exhibited higher incidences of all complications than did those without AKI, including mechanical ventilation, shock, bloodstream infection, intensive care unit (ICU) admission, and hemodialysis. The patients with AKI not only had more hospitalization days but also a significantly higher risk of in-hospital mortality and 1-year mortality.

**Table 2:** Disease details and outcomes at diagnosis of SJS, stratified by AKI Status.

Variable	All patients	AKI	Non-AKI	*P* value
Disease type, n (%)				0.007
SJS	56 (74.7)	12 (52.2)	44 (84.6)	
TEN	16 (21.3)	9 (39.1)	7 (13.5)	
Overlap syndrome	3 (4.0)	2 (8.7)	1 (1.9)	
Drug, n (%)				0.234
Allopurinol	24 (32.0)	10 (43.5)	14 (26.9)	
Phenytoin	10 (13.3)	3 (13.0)	7 (13.5)	
Carbamazepine	6 (8.0)	0 (0)	6 (11.5)	
Trimethoprim-sulfamethoxazole	3 (4.0)	1 (4.3)	2 (3.8)	
NSAIDs	1 (1.3)	1 (4.3)	0 (0)	
Others	31 (41.3)	8 (34.8)	23 (44.2)	
Complication, n (%)				
Mechanical ventilation	15 (20.0)	11 (47.8)	4 (7.7)	< 0.001
Shock	19 (25.3)	15 (65.2)	4 (7.7)	< 0.001
Bloodstream infection	8 (10.7)	6 (26.1)	2 (3.8)	0.009
Intensive care unit admission	23 (30.7)	15 (65.2)	8 (15.4)	< 0.001
Hemodialysis	14 (18.7)	14 (60.9)	0 (0)	< 0.001
Outcome, n (%)				
Hospital days	13 (11)	18 (28)	11 (8)	0.012
In-hospital mortality	15 (20.0)	13 (56.5)	2 (3.8)	< 0.001
1-year mortality	19 (25.3)	16 (69.6)	3 (5.8)	< 0.001

Continuous data are presented as median (interquartile range).

AKI, acute kidney injury; DRESS, drug reaction with eosinophilia and systemic symptoms; NSAIDs, nonsteroidal anti-inflammatory drugs; SJS, Stevens–Johnson syndrome; TEN, toxic epidermal necrolysis.

To evaluate the impact of AKI on in-hospital mortality, AKI was adjusted for in the univariable and multivariable models (Table 3). The presence of AKI was correlated with increased risk of in-hospital mortality in the unadjusted model (OR, 32.5 in Model 1). With adjustment for baseline factors such as age and sex in Model 2 and underlying diseases (i.e., diabetes and chronic kidney disease) in Model 3, the respective ORs were 23.9 and 18.4, both statistically significant. The association between AKI and risk of in-hospital mortality remained even after adjustment for disease type (OR, 9.2; 95% CI, 1.03–81.5). Notably, the 95% confidence intervals in Model 4 crossed 1, indicating that the model was overfitted. In addition, the 2-way interactions between AKI and other variables were not significant (data not shown).

**Table 3:** Association of AKI with risk of in-hospital mortality.

Model	OR	Empirical estimates: 95% CI of OR	Bootstrap estimates: 95% CI of OR
Model 1, unadjusted	32.5	6.3 to 166.9	9.0 to >10000
Model 2, adjusted for age and sex	23.9	4.2 to 136.1	4.7 to >10000
Model 3, further adjusted for diabetes and CKD	18.4	2.5 to 136.4	1.4 to >10000
Model 4, further adjusted for disease type	9.2	1.03 to 81.5	0.04 to >10000

AKI, acute kidney injury; CI, confidence interval; CKD, chronic kidney disease; OR, odds ratio.

**Fig 1:**
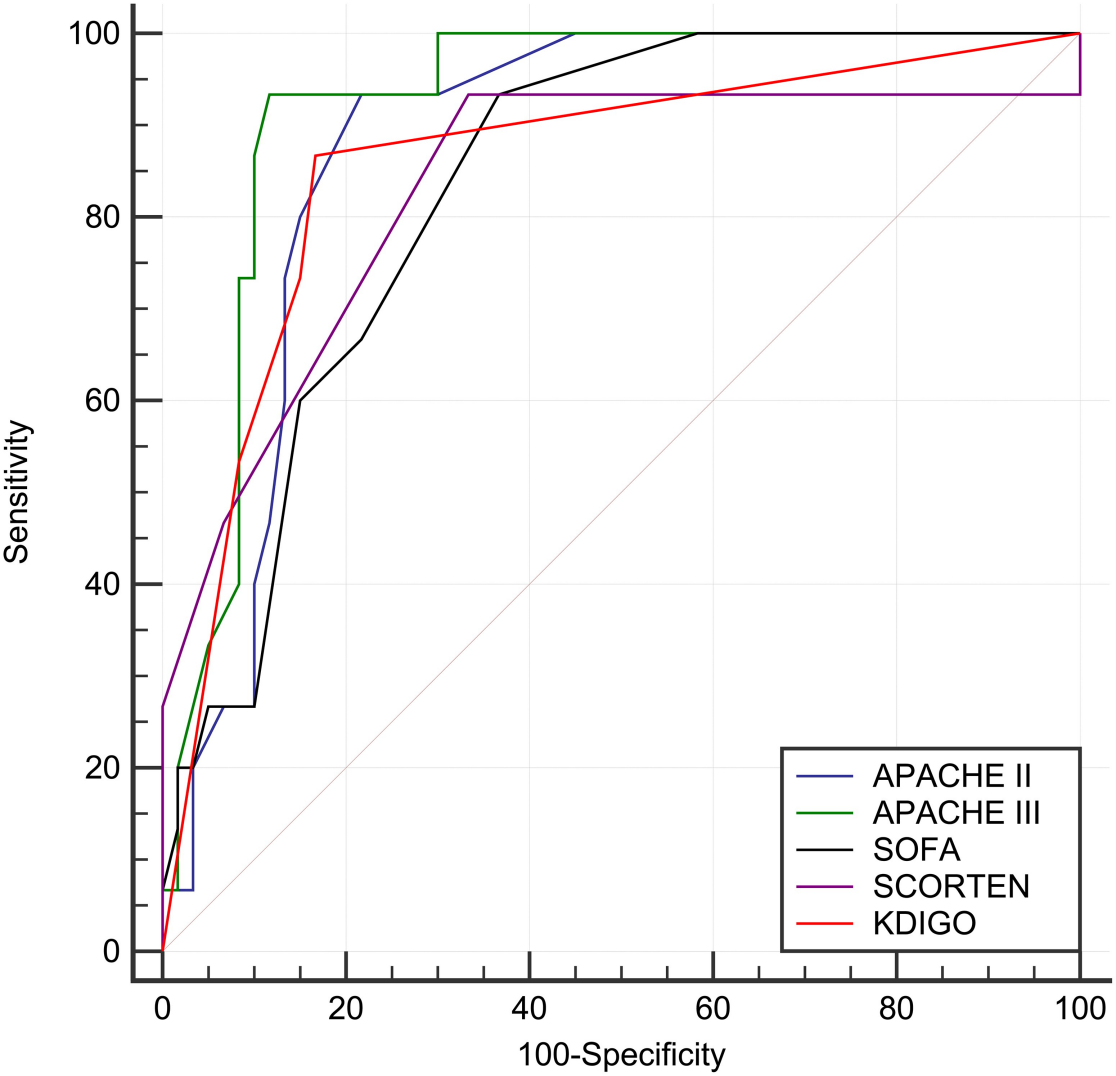
Discriminative ability of individual scores in predicting in-hospital mortality. The aura under the receiver operating characteristic curve (AUC) was 0.88 (95% CI 0.78 to 0.94), 0.92 (95% CI 0.84 to 0.97), 0.83 (95% CI 0.73 to 0.91), 0.83 (95% CI 0.73 to 0.91), and 0.86 (95% CI 0.76 to 0.93) for APACHE II, APACHE III, SOFA, SCORTEN, and KDIGO, respectively. Notably, the difference in AUC between KDIGO and the other prognostic scores was not significant (*P* = 0.686 for APACHE II, *P* = 0.250 for APACHE III, *P* = 0.677 for SCORTEN, and *P* = 0.715 for SOFA).

To compare the discriminative ability of the prognostic models and AKI severity, the AUCs were compared (Fig 1). The AUCs were 0.88, 0.92, 0.83, and 0.86 for APACHE II, APACHE III, SOFA, and KDIGO, respectively. Notably, the AUC for KDIGO was not significantly different from those for the other prognostic models (*P* = 0.686 for APACHE II, *P* = 0.250 for APACHE III, and *P* = 0.715 for SOFA). The Youden Index-determined cut-off points and the properties of discrimination ability are provided in Table 4. The optimal cut-off point for KDIGO stage was determined to be more than 1, with a sensitivity of 73.3% and a specificity of 85% for predicting in-hospital mortality. The *P* values of the Hosmer–Lemeshow goodness-of-fit test for APACHE II, APACHE III, SOFA, SCROTEN, and KDIGO were 0.101, 0.423, 0.021, 0.011, and 1.000, respectively, indicating that the KDIGO model was well calibrated (data not shown).

**Table 4:** Property of discriminative ability of individual scores in predicting in-hospital mortality.

Score	Cut-off#	Youden Index	Sensitivity (95% CI)	Specificity (95% CI)	+LR (95% CI)	-LR (95% CI)
APACHE II	> 9	0.72	89.5 (66.9–98.7)	82.1 (69.6–91.1)	5.0 (2.8–9.0)	0.13 (0.03–0.5)
APACHE III	> 39	0.80	94.7 (74.0–99.9)	85.7 (73.8–93.6)	6.6 (3.5–12.7)	0.06 (0.009–0.4)
SOFA	> 1	0.56	89.5 (66.9–98.7)	66.1 (52.2–78.2)	2.6 (1.8–3.9)	0.16 (0.04–0.6)
SCORTEN	> 2	0.60	93.3 (68.1–99.8)	66.7 (53.3–78.3)	2.8 (1.9–4.1)	0.1 (0.01–0.7)
KDIGO	> 1	0.70	73.3 (44.9–92.2)	85.0 (73.4–92.9)	4.9 (2.5–9.6)	0.3 (0.1–0.7)

+LR, positive likelihood ratio; -LR, negative likelihood ratio; APACHE, Acute Physiology and Chronic Health Evaluation; KDIGO, Kidney Disease Improving Global Outcomes; SCORTEN, severity-of-illness for toxic epidermal necrolysis; SOFA, sequential organ failure assessment score.

^#^ is according to Youden Index.

**Fig 2:**
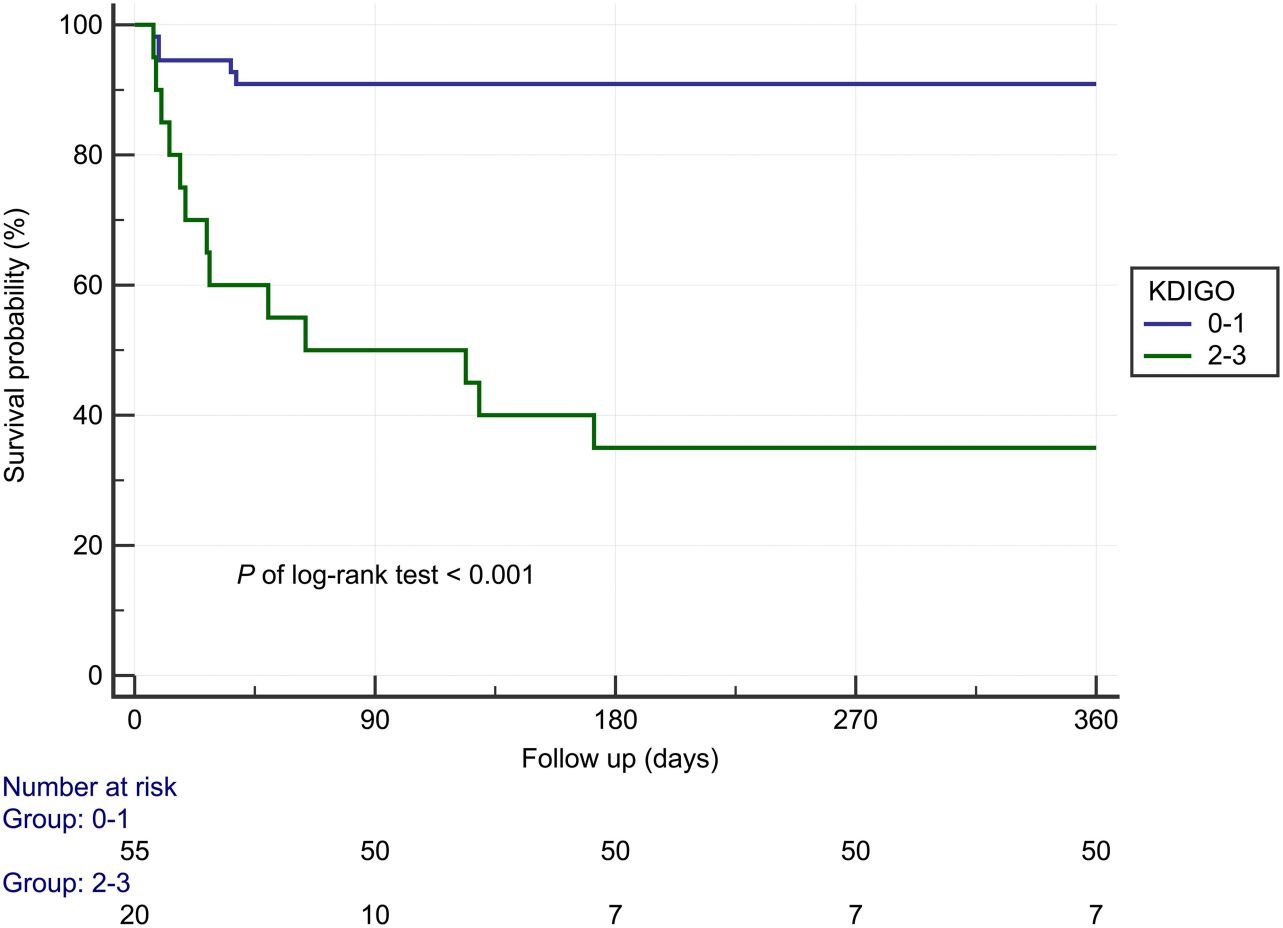
Survival curves of 1-year mortality stratified by KDIGO staging. KDIGO, Kidney Disease Improving Global Outcomes.

Fig 2 shows the 1-year Kaplan–Meier survival curves stratified by KDIGO stage (for stages 2 and 3), revealing that the patients with AKI stages 2 and 3 had significantly poorer outcomes compared to those without AKI or with AKI stage 1 (*P* < 0.001).

Table 5 summarizes the discrimination and reclassification results for combining KDIGO with other scoring systems in predicting in-hospital mortality. The results reveal that adding KDIGO resulted in greater discrimination ability (IDI) compared with using solitary APACHE II, solitary SOFA, and solitary SCORTEN. The results also show that KDIGO provided additional predictive ability (continuous NRI) beyond the individual scores.

**Table 5:** Properties of discrimination and reclassification for combining KDIGO with other individual models in predicting in-hospital mortality.

Model	IDI (95% CI)	*P* value	NRI (95% CI)	*P* value
APACHE II	15.9% (2.4%, 29.4%)	0.021	133% (89%, 177%)	<0.001
APACHE III	7.8% (-2.9%, 18.5%)	0.152	117% (68%, 165%)	<0.001
SOFA	20.7% (6.0%, 35.4%)	0.006	130% (86%, 174%)	<0.001
SCORTEN	14.2% (5.5%, 22.9%)	0.001	100% (49%, 151%)	0.0005

APACHE, Acute Physiology and Chronic Health Evaluation; CI, confidence interval; IDI, integrated discrimination index; KDIGO, Kidney Disease Improving Global Outcomes; NRI, net reclassification index; SCORTEN, severity-of-illness for toxic epidermal necrolysis; SOFA, sequential organ failure assessment score.

## Discussion

In the present study, of the 75 patients diagnosed with SJS/TEN between January 2011 and December 2014, 23 (36.0%) were diagnosed as having AKI at the same time. These patients were found to have significantly higher rates of in-hospital mortality and 1-year mortality compared with their non-AKI counterparts. The prevalence of drug-induced adverse effects was previously reported to be 1–6 cases/100 000 person-years among patients with SJS and 0.4–1.2 cases/100 000 person-years among those with TEN. [18] The RegiSCAR study found the mortality rate of severe drug-induced cutaneous adverse effects to be 23% at 6 weeks and 34% at 1 year. [5] Derek et al. reported in-hospital mortality of 4.8% for students with SJS, 19.4% for those with SJS/TEN, and 14.8% for those with TEN. [8] The in-hospital mortality rate of SJS/TEN has been reported to range from 26.7% to 32%, similar to our result. [12, 19, 20] Concerning AKI, a previous study reported its incidence among patients with SJS/TEN to be 20.8%. [7] Another study reported AKI prevalence of 2.7% among patients with SJS and 1.5% among those with SJS/TEN and TEN. [8] The higher prevalence of AKI in our study might be because our research applied the newest definition of AKI as defined by KDIGO. In addition, our data source was a tertiary care medical center treating patients referred from another hospital; presumably, such patients have more severe or refractory cases, which might also have contributed to the higher AKI rate. Regarding the offending drugs, the most common SJS/TEN-inducing medication in our study was allopurinol, followed by phenytoin and carbamazepine. These results are similar to those of a prior case–control study. [21] Furthermore, AKI has previously been recognized as an independent risk factor for death in patients with SJS/TEN. [8, 22]

The pathophysiology of AKI in SJS/TEN is complex. Studies have reported that SJS and TEN are associated with increased fluid loss from mucosal damage to the skin and gastrointestinal clinical features, such as abdominal cramps, severe exudative diarrhea, and bleeding, [23] as well as with a higher infection rate, all of which contributes to renal dysfunction. Hung et al. argued that sepsis and hypoalbuminemia are independent risk factors for AKI in these patients. [7] In fact, sepsis led to acute tubular necrosis in 27%–35% of hospitalized patients [24]; furthermore, hypoalbuminemia, which might be related to malnutrition and decreased effective intravascular volume, was a strong predictor of AKI in ICU patients. [25] Infection complications such as sepsis, bacterial, fungal and viral infections are increased in SJS/TEN cases because of the loss of skin integrity. [26] Regarding coexisting drugs inducing interstitial inflammation, acute interstitial nephritis was confirmed through renal biopsy. [27] Furthermore, Spanou et al., by applying in vitro and phenotypic analysis, revealed that drug-induced nephritis and drug-induced cutaneous hypersensitivity reaction share generally homogenous cytokine patterns in the drug-specific T-cell pathway. [28] Both studies have implied that the coexistence of these 2 diseases, SJS/TEN and AKI, is caused by similar offending drugs.

Concerning factors associated with mortality, Chung et al. reported that renal insufficiency as well as increased oxypurinol and granulysin levels were correlated with the poor prognosis of allopurinol-associated cutaneous adverse reaction. [22] Hsu et al. reported that age, number of chronic conditions, infections (i.e., septicemia, pneumonia, and tuberculosis), hematological malignancy (i.e., non-Hodgkin’s lymphoma and leukemia), and renal failure were prognostic risk factors for mortality. [8] However, only Sylvise et al.’s SCORTEN score has been developed to predict death in patients with TEN. SCORTEN uses age, presence of malignancy, tachycardia, epidermal detachment at admission, serum urea, glucose, and bicarbonate level to calculate a score reflecting the risk of death for patients with TEN (AUC = 0.82). [12] SCORTEN score was modified from Simplified Acute Physiology Score II score, which used serum urea level as parameter intead of sCr. Although serum urea shows some correlation with sCr but urea might be influenced by dietary protein intake, nitrogen metabolism, hepatic function and even diuretics. Thus, the AKI task force used sCr as a parameter to defined AKI. Moreover, the KDIGO system reflects the changes of sCr which might explain the additional effect on SCROTEN.

Our research utilized common prognostic models widely used in ICUs and compared them with the KDIGO score model. Our result showed that the KDIGO score has similarly accurate discriminative ability as the common prognostic models. To our knowledge, this is the first study to use only AKI as an outcome predictor and to use AKI severity according to the KDIGO scoring system as a prognostic model for SJS/TEN. Requiring only examination of changes in SCr and urine amount, the current approach might constitute a simple predictive tool for assessing patients with SJS/TEN. Since adding KDIGO staging significantly improved the discriminative power while using other prognostic models, we also suggested applying KDIGO staging system to identify patients with AKI in clinical practice.

The management of SJS/TEN in current clinical practice comprises early diagnosis and severity assessment, the prompt withdrawal of the offending agent, and supportive treatment. The main elements of such supportive care are similar to those for burn injuries and include wound care, fluid and electrolyte management, nutritional support, temperature management, pain control, monitoring or treating superinfections, and, if necessary, ICU or burn center admission. [29] Aggressive fluid and electrolyte management is emphasized due to increased water loss from the denuded dermis in SJS/TEN as well as to prevent AKI. One study reported that 2 mL/kg of body weight multiplied by the percentage of body-skin area skin detachment resulted in adequate urine output and significant correction of the base deficit in patients with TEN. [30] Recently, AKI care bundles have shown the ability to improve outcomes. [31–33] Our study results, with follow-up of up to 1 year, reveal that the patients with AKI stages 2 and 3 have a significantly inferior survival rate compared with the patients without AKI or with AKI stage 1. The further application of AKI bundles in patients with SJS/TEN who are diagnosed as having AKI warrants more investigation.

Despite the encouraging results of this study, a few limitations should be noted. First, this study used a retrospective design with data from a single tertiary care medical center, which might limit the generalizability of the findings. Second, the association of SJS/TEN with genes, ethnicity, and genetic variation should be considered when expanding our results to populations with different ethnic compositions. Third, our study was unable to examine all possible AKI etiologies of the patients—a factor that might have influenced their mortality rate. Finally, due to the low number of mortality events (n = 15) in this study, the problem of overfitting was encountered in the multivariable model; however, the rule of 10 events per variable may be relaxed in some situations. [34]

In conclusion, the SJS/TEN patients with AKI—particularly those with stages 2 and 3—exhibited significantly higher in-hospital mortality and 1-year mortality compared with those without AKI. The KDIGO staging provided a similar discriminative ability in predicting in-hospital mortality as did the APACHE II, APACHE III, SOFA, and SCORTEN scores. Furthermore, combining KDIGO with other scoring systems yielded significantly more accurate risk prediction for in-hospital mortality compared with the other individual scores alone, as measured by NRI. Further investigation should focus on the SJS/TEN interventions that bundle care once AKI is diagnosed.
